# Agreements and disagreements in exercise therapy prescriptions after hip replacement among rehabilitation professionals: a multicenter survey

**DOI:** 10.1186/s12891-015-0646-7

**Published:** 2015-08-05

**Authors:** Christine Eulenburg, Anna-Lina Rahlf, Andrej Kutasow, Astrid Zech

**Affiliations:** Department for Medical Biometry and Epidemiology, University Medical Center Hamburg-Eppendorf, Hamburg, Germany; Department of Exercise Physiology, Friedrich Schiller University of Jena, Jena, Germany; Institute of Human Movement Science, University of Hamburg, Hamburg, Germany

**Keywords:** Hip arthroplasty, Prescription standards, Guidelines, Postoperative care

## Abstract

**Background:**

Exercise therapy following total hip replacement (THR) is considered to be important during the initial postoperative care, but till date only a few evidence-based recommendations exist. The aim of this survey was to identify prescription standards among different rehabilitation professionals, for the exercise therapy management after THR in Germany.

**Methods:**

The study was a cross-sectional survey. Standardized questionnaires were sent to 38 eligible rehabilitation facilities in Germany. Participating surgeons, orthopaedic physicians, physiotherapists and exercise therapists rated the optimal early weight-bearing, resistance training, key components and dose of exercise therapy, and the hip loading during exercising. The returned questionnaires were then analyzed for level of agreement (≥80 %) among respondents.

**Results:**

313 rehabilitation professionals from 28 clinics returned completed questionnaires and were considered eligible for analysis. Out of total respondents, 53.9 % (cemented THR) and 18.2 % (uncemented THR) recommended full weight-bearing within five days after surgery. Commencement of resistance training later than three weeks after surgery is recommended by 20.6 % (36 %) for cemented (uncemented) prosthesis. Feedback varied significantly amongst the professions. Regarding the overall objectives of rehabilitation after hip replacement, respondents agree in six out of eight requested items. Agreement concerning priorities of specific exercises was achieved in three out of twelve items. The recommended exercise therapy dose varied significantly with working experience (p = 0.02).

**Conclusion:**

Rehabilitation professionals mainly disagreed with the exercise therapy prescriptions following the total hip replacement during the initial postoperative care in Germany.

**Electronic supplementary material:**

The online version of this article (doi:10.1186/s12891-015-0646-7) contains supplementary material, which is available to authorized users.

## Background

Total hip replacement (THR) is one of the most commonly practiced orthopaedic surgeries in developed countries [1]. According to the latest OECD survey (2013), Germany (after Switzerland) had the second highest rate of hip replacement in 2011 with 286 surgeries per 100.000 citizens [2]. The main proportion (more than 80 %) of THR patients suffered from osteoarthritis, followed by rheumatoid arthritis or osteonecrosis [1]. Hip replacements have been shown to reduce pain and disability effectively, and to improve quality of life [3, 4]. Nevertheless, during the initial postoperative phase THR patients are considerably limited in their normal function and mobility [5, 6]. Physical and exercise therapy approaches are widely accepted as treatment of choice in order to restore full mobility and physical functions in hip arthroplasty patients [7, 8]. However, although there is a common opinion for the importance of postoperative treatment in regaining physical functions [9, 10], only a few evidence-based recommendations exist for rehabilitation after THR [11, 12]. The systematic review of Di Monaco et al. [11, 12] demonstrated the convincing evidences for the effectiveness of treadmill training, quadriceps strength training and arm ergometry exercises. Benefits of other interventions or the possible superiority of individual exercise, however, mostly remain unclear. The research deficit could be explained with the lack of randomized controlled trials, comparing two or more exercise regimes regarding clinically relevant outcomes. Hence, it is not surprising that till date there are no international standards for the postoperative care in terms of (a) length of stay, (b) discharge disposition to home or inpatient rehabilitation and (c) timing of rehabilitation [1]. According to the results from the Global Orthopaedic Registry (GLORY) [1], the total length of hospital stay after THR is three days in the US, nine days in the UK, eleven days in Germany and 30 days in Japan, respectively. In Germany, less than fifty percent of all patients are discharged directly to a rehabilitation hospital. A typical rehabilitation program begins a few days after hospital discharge, lasts between 2 and 4 weeks. It focuses on individual and group exercise therapy as well as functional exercises [13]. Best practice recommendations following THR in the US and Canada [14] include functional exercises (strengthening, active range of motion, balancing and stair climbing) and gait training. Two recently performed surveys among physiotherapists in the UK [11] and Netherlands [14], regarding rehabilitation practice standards emphasize the importance of functional, muscle strengthening, gait and active range of motion exercises. These surveys certainly provide important information on widely used components, yet no recommendations or standards could be found in the literature regarding the optimal dose of exercise therapy in the first postoperative weeks.

An increasing number of authors complain that current rehabilitation practice seems to be more guided by personal and institutional (rehabilitation setting) factors rather than by scientific findings [11, 15, 16]. This indicates that experience and preferences of surgeons, orthopaedic physicians and therapists play a major role in the postoperative management. But still no information has been collected on exercise therapy standards in various rehabilitation settings amongst the rehabilitation professionals. The investigation of individual opinions and prescriptions may help in better understanding existing standards that influence physicians’ and therapists’ decision making, in planning and execution of exercise therapy interventions. It would also be an approach to assess the general compliance of the existing guidelines as well as to define relevant research questions for future studies in this field.

The objective of this multicenter survey was to identify prescription standards and personal beliefs regarding the optimal exercise therapy treatment for inpatient rehabilitation, after total hip replacement in Germany. It is hypothesized that rehabilitation professionals and institutions differ regarding exercise therapy prescriptions, and in their viewpoint on effective exercise measures and the optimal load on the joint during initial postoperative period.

## Methods

### Study design

The study was a cross-sectional survey of orthopaedic rehabilitation care professionals in Germany, conducted between November 2012 and November 2013.

### Participants, recruitment and setting

The orthopaedic rehabilitation centres participating in the study were recruited using a database of Germany-wide rehabilitation facilities [17]. All facilities listed in the field of orthopaedic diseases were considered to be potentially eligible and were included in the initial recruitment stage. Clinics focusing on other medical conditions were excluded. A search option within the website was used to identify facilities providing orthopaedic rehabilitation treatment. No search limits were defined regarding geographical locations or for the clinic type (inpatient/outpatient). Potentially eligible facilities were contacted by email and asked if they provided rehabilitation treatment for THR patients, and if they were interested in participating in the survey. The email contained information on the study purpose and methods as well as an example of the questionnaire. Facilities keen in participation were then requested to send their feedback on the number of questionnaires they needed. After receiving their acceptance a formal letter with formal instructions to be followed and the requested number of questionnaires were sent to the clinics. Facilities were requested to distribute the questionnaires together with an informational letter among all surgeons, orthopaedic physicians, physiotherapists and exercise therapists who were involved in hip arthroplasty rehabilitation. If the participating facility showed reluctance in sending the duly filled-in questionnaire within three months, then a reminder email was sent. The questionnaire is published as additional file in the original German version (e.g. Additional file 1) and in a translated English version (e.g. Additional file 2).

### Procedure

The questionnaire was developed according to the current standards of exercise therapy in western countries [16] and Germany in particular [13]. It included four categories with overall 14 questions to be answered. Questions generation was based on a brief literature review and valuable discussions with the experts. Prior to its distribution to the participating centers, the questionnaire was pilot-tested in two different rehabilitation centers. Results from these centers indicated that the majority of participants understood the questions and response options. The internal consistency of the questionnaire was found to be good (Cronbach’s alpha: 0.77). In the first part, data on the professional background were assembled, which included the profession, working experience in years, type of facility and average number of THR patients per month. The second part contained general questions on rehabilitation. Participants were asked to rate the importance of influencing factors as per priority from one (not important) to five (very important). Additionally, participants were asked to suggest the optimal time for starting full weightbearing and resistance training after cemented and uncemented hip replacement from a selection of seven predefined time intervals as stated in the questionnaire for full weightbearing and five intervals for therapy start. In the third part, participants were asked to rate the facility-specific priorities of existing exercise therapy measures (e.g. group exercise, continuous passive motion) and therapy objectives (e.g. pain reduction, strengthening of hip muscles) from one (highest priority) to five (lowest priority). Further, ratings regarding optimal strength training intensity at 15 days and three months postoperative were assessed using the Borg “rating of perceived exertion” (RPE) scale ranging from six to 20 [18]. The fourth part contained items on estimated joint loading. For selected types of exercises or motion (e.g. walking, stair climbing, cycling), participants were asked to estimate the hip joint loading between 1 (very low stress) and 10 (maximum stress).

### Data analysis and statistics

Descriptive statistics were used to evaluate demographic data. Continuous variables were reported as mean (standard deviation) and categorical variables were presented as frequencies (n) and percentages (%). Agreement of the respondents concerning specific items of the questionnaire was the primary outcome. Questions on personal opinions on the importance of influencing factors on the postoperative rehabilitation treatment and on clinic-specific goals and measures of training therapy were assessed on five-point Likert scales. To define agreement for the item on the importance of influencing factors for the treatment, we condensed values 1 and 2 into “not important”, value 3 represents “medium important” and 4 and 5 combined to “important”. Regarding facility-specific goals and measures, we condensed values 1 and 2 into “high priority” and 4 and 5 into “low priority”. Value 3 represents “medium priority”. Analogously, the ten-point Likert scales assessing personal opinions on joint load of specific exercises were recoded into three categories. Values 1–3 represent “low stress”, 4–7 “intermediate stress” and 8–10 “high stress”, respectively. Agreement was defined, when more than 80 % of answers coincided in one of the three combined categories. This technique of measuring agreement has been previously reported [19–21]. Marx et al. [19] used 80 %, while Wright et al. [20] used 90 % as cut-off value for agreement. Mamlin et al. [21] defined agreement, if more than 60 % of the raters chose the combined category. We are in consonance with Marx et al. [19], indicating 90 % too strict, while 60 % may overstate agreement and chose 80 % as cut-off value for agreement. However, the percentages are cited in this work, so that the reader is free to choose his own critical value. Absolute and relative frequencies regarding the favoured time points of full weight-bearing and of therapy start after surgery have been reported. The Borg RPE scales were analyzed as continuous variables. For statistical inference, tests were performed to detect if systematic differences between professions or clinic types are present with regards to all items. Linear mixed models were adapted including profession, professional experience, type of clinic and number of THR patients within the clinic as fixed covariables. To consider that subjects within one clinic may be more related than subjects across different clinics, the institution was included as a random effect. For this analysis, original values of the categorical Likert scales were used as continuous dependent variables. Instead of the predefined time intervals concerning start of full weight-bearing and of therapy after surgery mean values of time intervals were used for significance tests. The significance level was set to 5 %. SPSS version 21.0 for Windows (SPSS, Chicago, Illinois, USA) was used for statistical analysis. [22]

#### Ethics

The questionnaire used for the survey was anonymous. No personal data were collected from respondents. A statement at the title page of the questionnaire included information regarding the study purpose and the anonymity of responses. The response to the survey was assumed to indicate their informed consent. As the study concerned a survey to be completed anonymously and once only, no review by the Medical Ethical Committee of the University was needed. This procedure was in agreement with previous studies using similar research designs in this research field [14]. The study was conducted in accordance with the Handbook for Good Clinical Research Practice of the World Health Organization. The ethical principles of the Declaration of Helsinki were followed.

## Results

**Fig. 1:**
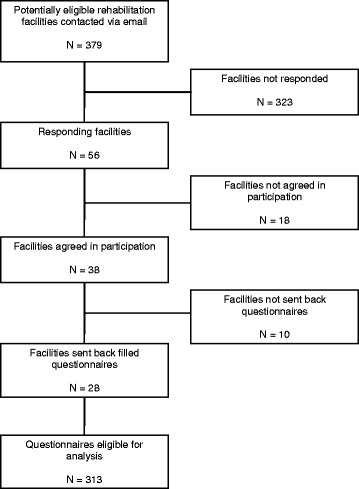
Flow chart showing inclusion and exclusion criteria for eligible rehabilitation facilities

After the initial database search, 379 potentially eligible rehabilitation facilities in Germany were contacted. From the 56 responding facilities (14.8 %), 38 agreed and were considered eligible, and received questionnaires. 28 clinics (73.7 %) located in 11 of the 16 states in Germany returned duly filled in 313 questionnaires of 168 (53.7 %) physiotherapists, 43 (13.7 %) rehabilitation physicians, 18 (5.8 %) orthopaedic surgeons and 84 (26.8 %) exercise therapists. A few clinics declined participation via telephone or email due to lack of time or lack of personals who agreed to complete the questionnaire. Other clinics did not respond to the initial or reminder e-mail. All 313 returned questionnaires were analyzed. A flowchart displaying the recruitment process is shown in Fig. 1. A detailed description of the participants is given in Table 1.

**Table 1:** Descriptive statistics of the 313 participating rehabilitation professionals

	n	Percent	
**Professional group**			
Exercise therapists	84	26.8	
Surgeons / orthopaedic physicians	18	5.8	
Rehabilitation physicians	43	13.7	
Physiotherapists	168	53.7	
**Facility type**			
Outpatient rehabilitation clinics	8	2.6	
Hospitals	25	8.0	
Inpatient rehabilitation clinics	277	88.5	
	**mean**	**range**	**Inter-quartile range**
**Work experience (years)**	13	<1–43	4.25–20
**Number of THR patients per month**	120	1–300	6–25

### Personal opinion regarding the optimal treatment

Participants agreed that the course and the quality of surgery as well as the constitution of the patients individually have a major impact on the postoperative rehabilitation treatment. Disagreement was assessed regarding the impact of the type of prosthesis for primary or secondary hip replacement (Table 2). Results concerning the recommended start of full weight-bearing and resistance training after implantation are shown in Table 3. For cemented prosthesis, approximately half of the participants recommended a time between 0–5 days. For uncemented prosthesis, the majority of respondents declared that full weight-bearing should not start before the 10^th^ day post-surgery. Disagreements were also observed for the best time to start resistance training. One fifth and one third of the respondents recommended to wait for more than three weeks following surgery for cemented and uncemented prostheses, respectively. The distribution of answers differed significantly between professions for cemented (p = 0.03) and uncemented (p < 0.001) prostheses, adjusted for professional experience, clinic type and number of THR patients per month. Surgeons recommended an early start of full resistance training as compared to therapists and rehabilitation physicians. Estimated means (confidence intervals CI) for surgeons were 12.7 days (95 % CI [5.2; 20.2]) and 14.8 days (95 % CI [5.9; 23.7]) for cemented and uncemented prostheses, respectively. In contrast, estimated means for physiotherapists were 20.1 days (95 % CI [14.8; 25.3]) and 27.7 days (95 % CI [21.3; 34.0]), respectively.

**Table 2:** Personal opinion on the importance of factors influencing the postoperative rehabilitation treatment. Agreement is defined when more than 80 % of answers coincide in one of the three combined categories

	*Missing*	*Not important*	*Medium important*	*Important*
	*n (%)*	*n (%)*	*n (%)*	*n (%)*
**Agreement**				
Course of surgery	10 (3.2)	13 (4.4)	39 (12.9)	251 (80.2)
Quality of surgery	7 (2.2)	8 (2.6)	30 (9.8)	268 (87.6)
Constitution of the patients	19 (6.1)	10 (3.4)	28 (9.5)	256 (87.1)
**Disagreement**				
Type of prosthesis (cemented / uncemented)	9 (2.9)	120 (39.5)	88 (28.9)	96 (31.6)
Primary or secondary hip replacement	7 (2.2)	25 (8.2)	69 (22.5)	212 (69.3)

**Table 3:** Personal recommendations regarding the optimal time to start full weight-bearing and resistance training following total hip replacement

	Cemented prosthesis		Uncemented prosthesis	
	n	%	n	%
**Full weight-bearing**				
0–5 days	166	53.9	55	18.2
6–10 days	72	23.4	54	17.9
11–20 days	35	11.4	61	20.2
21–30 days	20	6.5	77	25.5
5–6 weeks	9	2.9	35	11.6
7–8 weeks	6	1.9	14	4.6
>8 weeks	0	0.0	6	2.0
Missing information	5	1.6	11	3.5
**Resistance training**				
1–7 days	60	19.6	41	13.5
2–3 weeks	183	59.8	153	50.5
4–5 weeks	46	15.0	68	22.4
6–7 weeks	8	2.6	22	7.3
>8 weeks	9	2.9	19	6.2
Missing information	7	2.2	10	3.2

### Facility-specific objectives and key components of rehabilitation practice

Regarding the overall objectives of rehabilitation after hip replacement predefined by the clinics respondents agreed that postoperative treatment should focus on improving mobility, gait, daily activities, hip strength to reduce pain and muscle imbalance (between 82.5 and 87.7 %). Disagreements were observed regarding the importance of balance control and core stability (Table 4). When asked for the clinic-specific key components of exercise therapy, participants agreed that gait and stair climbing exercises as well as individual physiotherapy should be performed with the highest priority. Other components (e.g. continuous passive motion, neuromuscular training, water exercises) were rated with a different priority among respondents (Table 4). The priorities of water exercises and ergometer cycling differed with facility types. Professionals working in hospitals rated water exercises with a higher priority (p = 0.01) as compared to the other respondents. Furthermore, those who worked in inpatient rehabilitation clinics rated the priority of ergometer cycling lower than the other participants (p = 0.01).

**Table 4:** Facility-specific overall objectives for the rehabilitation of THR patients and key exercise therapy components are shown. Agreement is defined when more than 80 % of answers coincided in one of the three combined categories

	*Missing*	*Low priority*	*Medium priority*	*High priority*
**Objectives**	*n (%)*	*n (%)*	*n (%)*	*n (%)*
**Agreement**				
Reducing muscular imbalances	9 (2.9)	29 (9.5)	17 (5.6)	258 (84.9)
Improving mobility	7 (2.2)	30 (9.8)	12 (3.9)	264 (86.3)
Restoring functional gait patterns	7 (2.2)	29 (9.5)	9 (2.9)	268 (87.6)
Recovery of activities of daily living	7 (2.2)	31 (10.1)	23 (7.5)	252 (82.4)
Pain reduction / freedom of pain	5 (1.6)	30 (9.7)	24 (7.8)	254 (82.5)
Strengthening of hip muscles	3 (1.0)	31 (10)	7 (2.3)	272 (87.7)
**Disagreement**				
Improving balance control	13 (4.2)	26 (8.6)	51 (17.0)	223 (74.3)
Improving core stability	13 (4.2)	24 (8.0)	82 (27.3)	194 (64.7)
**Key exercise therapy components**				
**Agreement**				
Gait training	7 (2.2)	26 (8.5)	12 (3.9)	268 (87.6)
Stair climbing	12 (3.8)	27 (8.7)	24 (7.7)	250 (83.1)
Individual physiotherapy	7 (2.2)	28 (9.1)	8 (2.6)	270 (88.2)
**Disagreement**				
Gym exercises	13 (4.2)	40 (13.4)	66 (22.0)	194 (64.7)
Continuous passive motion	31 (10.0)	110 (39)	60 (21.3)	112 (39.7)
Neuromuscular /sensorimotor training	37 (11.8)	39 (14.1)	64 (23.2)	173 (62.7)
Stretching	21 (6.7)	42 (14.4)	51 (17.5)	199 (68.2)
Water exercises	12 (3.8)	36 (12.0)	49 (16.3)	216 (71.8)
Ergometer cycling	19 (6.1)	52 (17.6)	77 (26.2)	165 (56.1)
Walking exercises	38 (12.1)	149 (54.1)	63 (22.9)	63 (22.9)
Manual therapy	31 (10.0)	83 (29.5)	58 (20.6)	141 (50.0)
Group exercises	16 (5.1)	40 (13.5)	36 (12.1)	221 (74.4)

### Exercise therapy dose

Participants were also asked for their personal opinions about what the perceived exertion in lower extremity resistance training should be 15 days and 3 months after THR. The Borg RPE scale after 15 days had a mean value of 12.6 (SD 1.9), indicating a “fairly light” to “somewhat hard” intensity. The rating was significantly influenced by the years of working experience (p = 0.02). One year working experience decreased the mean reported RPE by 0.03 (95 % CI [0.00; 0.06]). After 3 months, the optimal resistance training intensity was considered as mean RPE of 13.7 (SD 2.5). The profession had a significant impact on the reported RPE (p = 0.03). Rehabilitation physicians indicated the lowest estimated mean RPE (12.2 (95 % CI [10.9; 13.4]) “fairly light” to “somewhat hard”), while physiotherapists and exercise therapists had the highest mean RPE value (13.5 (95 % CI [12.5; 14.5]) “somewhat hard”).

### Hip joint load during exercise therapy

Therapists were asked to rate the hip joint load during specific exercises on a scale from one to ten. Mean values (standard deviations) for ergometer cycling, low speed, high resistance were 3.2 (1.7) and 6.5 (2.1), while for walking, standing on one leg and sitting, mean estimates were 6.0 (2.1), 7.9 (2.1) and 4.3 (2.1), respectively. Furthermore, standing up, bridging and abduction in lateral position yielded mean values (SD) of 6.5 (2.1), 5.9 (2.2) and 6.6 (2.2). Table 5 represents the agreement concerning the hip joint loading during specific exercises, divided into three categories low, medium and high stress. No agreement was achieved across respondents regarding any of the exercise components

**Table 5:** Agreement concerning joint load of the hip joint loading during specific exercises is shown

	*Missing*	*Low loading*	*Medium loading*	*High loading*
	*n (%)*	*n (%)*	*n (%)*	*n (%)*
**Disagreement**				
Ergometer cycling, low resistance	10 (3.1)	199 (65.7)	96 (31.7)	8 (2.6)
Ergometer cycling, high resistance	12 (3.8)	26 (8.6)	157 (52.2)	118 (39.2)
Walking 4 Km/h	13 (4.2)	40 (13.3)	176 (58.7)	84 (28.0)
One-leg standing	9 (2.9)	16 (5.3)	91 (29.9)	197 (64.8)
Sitting	11 (3.5)	129 (42.7)	146 (48.3)	27 (8.9)
Chair rise	9 (2.9)	24 (7.9)	177 (58.2)	103 (33.9)
Bridging	15 (4.8)	53 (17.8)	171 (57.4)	74 (24.8)
Abduction in lateral position	12 (3.8)	31 (10.3)	158 (52.5)	112 (37.2)

## Discussion

This survey presents current prescription standards and personal beliefs regarding the optimal exercise therapy treatment for inpatient rehabilitation after hip replacement in Germany. While previous surveys [11, 14] in this field mainly have focused on the importance of single exercise regimes, the present study provides further investigations on the optimal dose (intensity) as well as the estimated hip joint loading during specific exercises.

The main finding of this study was that all respondents agreed on the general objectives of rehabilitation, following THR but disagreed concerning the timing, dose and components of exercise therapy during the first few postoperative weeks.

### Agreements and disagreements in exercise therapy prescriptions

Considerable variations among respondents were found regarding the recommended beginning of full weight-bearing and resistance training after cemented and uncemented hip replacement. Till date no other surveys have collected the postoperative data on practice standards for these two milestones of postoperative treatment in THR patients. According to the current evidence [23–26], immediate full weight-bearing is feasible, not only for cemented but also for uncemented hip replacements. Buehler et al. [27] also showed that patients with delayed weight-bearing have an increased risk of deep venous thrombosis. However, in rehabilitation practice, immediate weight-bearing after uncemented THR is still controversial, due to fear of overloading the hip joint, which may negatively influence the ingrowth of the implant [28]. The results of our survey indicate that these concerns may still play a major role during rehabilitation as more than half of all respondents preferred partial weight-bearing during the first ten days after cementless replacement.

Regarding the beginning of full weight-bearing and resistance training, varying preferences were observed amongst different professions. Strengthening of lower extremity muscles is one of the major goals of rehabilitation following THR [14, 16]. Although a few studies indicated that an early maximum strength training is feasible and beneficial in these patients [29], the adequate intensity during the first postoperative weeks has also been controversially discussed. The observed differences between professions seem to reflect this uncertainty and emphasize the imperative need of evidence-based exercise guidelines for the first phase of postoperative treatment.

Agreement was found on the importance of gait training, stair climbing and individual physiotherapy. Comparable findings were reported in two recent surveys among rehabilitation professionals in the UK [11] and Netherlands [14]. Both these studies support the relevance of strengthening, functional and gait exercises for postoperative treatment. Consensus statements of rehabilitation expert panels in the US and Canada [16] also considered functional exercises (strengthening, active range of motion, balancing, stair climbing and gait training) are essential after primary THR. Nevertheless, only a few randomized controlled trials examined the effects of single exercise interventions early postoperatively [30–32] and to date, no evidence exists on the superiority of a specific exercise intervention [7, 8, 12].

Respondents were also asked to express their beliefs regarding the optimal lower extremity resistance training intensity after hip replacement. Dose–response effects of postoperative exercise therapy have been rarely investigated [8, 12] and no practical standards or evidence-based recommendations can be found in the literature. Consequently, exercise intensities during rehabilitation are mainly influenced by personal experience and beliefs of therapists. The mean recommended intensity at 15 days post-surgery (12.6 on Borg’s 6–20 RPE scale) has been considered fairly light to moderate [33]. Respondents with longer working experience preferred a more conservative therapy with lower perceived exertions, as compared to those with less working years. Considering that a RPE of 12–13 is a critical value for detectable exercise therapy effects [34] one may speculate that intensities below this point are probably insufficient for meaningful strength improvements. This implies that future research should not only focus on the “yes” or “no” for the application of single interventions but also on the intensity (or challenge) at which an exercise is sufficiently effective or optimal for functional improvements.

The survey also revealed uncertainty among rehabilitation professionals on the hip joint loading during walking, standing, cycling, stair climbing, chair rises, bridging and hip abduction. Only few studies with small sample sizes investigated the force acting on the hip during different activities [35–38]. They showed that normal walking increases the resultant hip joint force between two and three times of the body weight [35, 37, 38]. Comparable increments of peak hip contact forces were reported for stair climbing, one-leg standing and bridging exercises, whereas dynamic hip abduction and chair rise exercises resulted in lower loadings [36]. In consideration to these findings, our survey indicates that hip joint loading during exercising is often misjudged by therapists. This may consequently influence the therapist’s treatment approach and could reduce the efficacy of postoperative exercise therapy interventions.

### Study limitations

Several limitations should be considered for the final interpretation of results. From 379 contacted rehabilitation centers, 56 (14.8 %) expressed their interest to participate and were assessed for eligibility. Although no data is available for non-responding facilities, there could be several reasons that may have contributed to the low response rate. Firstly, an email address obtained from an online database was used to contact rehabilitation centers. No information was available, to confirm whether the request was actually forwarded to the head physician or to some other responsible personal. Further, it may have appeared that, due to lack of experience in the treating arthroplasty patients, some of the non-responding centers did not feel adequately qualified and confident to participate in the survey (e.g. orthopaedic diseases only as secondary diagnosis). Other potential reasons included the lack of interest as well as concerns regarding the dissemination of internal data. Finally, 28 rehabilitation centers returned 313 completed questionnaires, yielding a response rate of 7.4 %.

It should also be noted that the results are limited to German rehabilitation settings. The postoperative management of total hip replacement is influenced by regional health care systems and therefore, can not be compared with various other states [1, 39]. A typical German rehabilitation is performed in an in- or outpatient rehabilitation clinic and starts a few days after hospital discharge. Final decisions on post-operative treatment, start of full weight-bearing, maximum hip joint loading or possible restrictions during exercising are primarily the responsibility of orthopedic surgeons or rehabilitation physicians. Physiotherapists are not autonomous in their decision-making.

In addition, the questionnaire was developed exclusively for the present survey and was pre-tested as well as adjusted by a few orthopedic physicians, physiotherapists and exercise therapists. Hence, the chosen categories of the questionnaire and response options provided are partly influenced by personal experience and do not fully exclude the possibility of being biased.

### Implications for practice

Our survey suggests that decisions during rehabilitation practice following hip replacement in Germany are strongly influenced by personal experience and opinions and may vary largely among rehabilitation professionals. The observed lack of consensus regarding the most beneficial exercise therapy treatment observed may partly be due to substantial research deficit in this field [7, 8, 12]. However, this survey also suggests that existing research findings have not been sufficiently implemented into daily treatment practice of therapists. This includes knowledge on hip joint loadings during exercising and weight-bearing as well as effective components and doses of exercise therapy. Therefore, concepts, strategies and measures are needed to improve the transfer of evidence-based knowledge into rehabilitation practice for the postoperative management of hip replacements.

## Conclusion

In conclusion, this survey reveals substantial disagreements among rehabilitation professionals regarding exercise therapy prescriptions during the first postoperative weeks after hip replacement in Germany. Surgeons and therapists differ in their recommendations on weight-bearing and resistance training. Physiotherapists and exercise therapists prefer a more conservative approach with a delayed start of weight-bearing and resistance training. This is in contrast to current evidence and might be explained with more extensive interactions between patient and therapist during individual treatment sessions. Furthermore, the beliefs and prescriptions of respondents regarding the estimated hip loading are only partly in agreement with reported true loadings in the literature. Further investigation should explore the influence of professional groups and facilities on THR rehabilitation. More evidence-based recommendations on beneficial exercise therapy dosages and components are needed in order to define reasonable guidelines and standards for postoperative treatment.

## Additional files

Additional file 1:
**Original questionnaire.** (PDF 174 kb) Additional file 2:
**Translated questionnaire (English).** (PDF 157 kb)

## Abbreviations

THR: Total hip replacement
RPE: Rating of perceived exertion
