# Supplementary material for: Agreements and disagreements in exercise therapy prescriptions after hip replacement among rehabilitation professionals: a multicenter survey
Source: BMC Musculoskelet Disord. 2015 Aug 5;16:185. doi: 10.1186/s12891-015-0646-7 (PMC4526287; doi:10.1186/s12891-015-0646-7)
Supplement: Additional file 1: — Original questionnaire. (PDF 174 kb) [file 12891_2015_646_MOESM1_ESM.pdf]

Fragebogen zur bewegungstherapeutischen Verordnung nach Implantation einer Endoprothese am Hüftgelenk

Liebe Teilnehmer,
für die Erfassung von persönlichen Überzeugungen bzw. institutionellen Richtlinien bei bewegungstherapeutischen Behandlungen nach Implantation einer Endoprothese am Hüftgelenk, möchten wir Sie bitten folgende Fragen zu beantworten. Ihre Angaben werden uns helfen, aktuelle Standards von Nachbehandlungen in der Therapiepraxis zu erfassen und den möglichen Bedarf an weiteren Handlungsrichtlinien zu klären.
Alle Angaben werden selbstverständlich anonymisiert und vertraulich behandelt. Wenn Sie sich bei einer Frage unsicher sind, wählen Sie bitte die Antwort, die Ihrer Meinung nach am besten auf Sie zutrifft.
Vielen Dank für Ihre Mühe.

1. Personenbezogene Angaben (Zutreffendes bitte ankreuzen bzw. Ausfüllen)

1.1. Welcher Berufsgruppe gehören Sie an?

a. Chirurg(in) ☐
b. Orthopäd(in) ☐
c. Arzt/Ärztin in einer Rehabilitationseinrichtung ☐
d. Sporttherapeut(in) ☐
e. Physiotherapeut(in) ☐
f. Andere (bitte angeben) …………………………………………

1.2. Wie viele Jahre Berufserfahrung haben Sie? ………………

1.3. In welcher der folgenden Einrichtung sind Sie derzeit tätig?

a. Tagesklinik oder Privatpraxis ☐
b. Krankenhaus ☐
c. Rehabilitationseinrichtung ☐
d. Physiotherapeutische Praxis ☐
e. Andere (bitte angeben) ………………………………………..

1.4. Wie viele Patienten mit Implantation einer Endoprothese am Hüftgelenk behandeln Sie durchschnittlich im Monat?

…………..

2. Allgemeine Fragen zur Rehabilitation und Bewegungstherapie nach Implantation einer Endoprothese am Hüftgelenk (Zutreffendes bitte ankreuzen bzw. Ausfüllen)

2.1. Was hat Ihrer persönlichen Einschätzung nach Einfluss auf die postoperativen Reha-Maßnahmen? (Bitte kreuzen Sie auf der Skala von 1 (unwichtig) bis 5 (sehr wichtig) an, Mehrfachantworten möglich)

	**1**	**2**	**3**	**4**	**5**
a. Prothesenart (zementiert/zementfrei)	☐	☐	☐	☐	☐
b. Erstversorgung bzw. Zweitversorgung	☐	☐	☐	☐	☐
c. Qualität des chirurgischen Eingriffs	☐	☐	☐	☐	☐
d. OP-Verlauf	☐	☐	☐	☐	☐
e. Konstitution des Patienten	☐	☐	☐	☐	☐
Weitere mögliche Faktoren (bitte benennen):					
……………………………………….	☐	☐	☐	☐	☐
……………………………………….	☐	☐	☐	☐	☐
……………………………………….	☐	☐	☐	☐	☐
……………………………………….	☐	☐	☐	☐	☐

2.2. Ab wann sollte nach Ihrer Einschätzung, eine Vollbelastung nach Implantation einer **zementierten** Prothese bei komplikationsfreien Operationsverlauf postoperativ empfohlen werden?

**0-5 Tage**	**6-10 Tage**	**11-20 Tage**	**21-30 Tage**	**5-6 Wochen**	**7-8 Wochen**	**mehr als 8 Wochen**
☐	☐	☐	☐	☐	☐	☐

2.3. Ab wann sollte nach Ihrer Einschätzung, eine Vollbelastung nach Implantation einer **nicht-zementierten** Prothese bei komplikationsfreien Operationsverlauf postoperativ empfohlen werden?

**0-5 Tage**	**6-10 Tage**	**11-20 Tage**	**21-30 Tage**	**5-6 Wochen**	**7-8 Wochen**	**mehr als 8 Wochen**
☐	☐	☐	☐	☐	☐	☐

2.4. Wann sollten Ihrer Einschätzung nach medizinische Trainingstherapie (keine physiotherapeutische Akutversorgung) nach Implantation einer **zementierten** Prothese bei komplikationsfreien Operationsverlauf idealerweise beginnen?

**1-7 Tage postoperativ**	**2-3 Wochen postoperativ**	**4-5 Wochen postoperativ**	**6-7 Wochen postoperativ**	**mehr als 8 Wochen postoperativ**
☐	☐	☐	☐	☐

2.5. Wann sollten Ihrer Einschätzung nach medizinische Trainingstherapie (keine physiotherapeutische Akutversorgung) nach Implantation einer **nicht-zementierten** Prothese bei komplikationsfreien Operationsverlauf idealerweise beginnen?

**1-7 Tage postoperativ**	**2-3 Wochen postoperativ**	**4-5 Wochen postoperativ**	**6-7 Wochen postoperativ**	**mehr als 8 Wochen postoperativ**
☐	☐	☐	☐	☐

3. Fragen zu Inhalten und Zielen der Rehabilitation:

3.1. Welche Maßnahmen werden in Ihrer Einrichtung (mit welcher Priorität) in der Standard-Rehabilitation von Hüft-TEP Patienten durchgeführt. Bitte kreuzen Sie auf der Skala von 1 (höchste Priorität) bis 5 (niedrigste Priorität) an.

	**1**	**2**	**3**	**4**	**5**
a. MTT Gerätegestützt	☐	☐	☐	☐	☐
b. Motorschiene	☐	☐	☐	☐	☐
c. Sensomotorisches Training	☐	☐	☐	☐	☐
d. Dehnung	☐	☐	☐	☐	☐
e. Wassergymnastik	☐	☐	☐	☐	☐
f. Gangschulung	☐	☐	☐	☐	☐
g. Treppentraining	☐	☐	☐	☐	☐
h. Fahrradergometer	☐	☐	☐	☐	☐
i. Walking	☐	☐	☐	☐	☐
j. Manuelle Therapie	☐	☐	☐	☐	☐
k. Einzel KG	☐	☐	☐	☐	☐
l. Gruppen KG	☐	☐	☐	☐	☐

3.2. Welche Ziele werden in Ihrer Einrichtung (mit welcher Priorität) in der Standard-Rehabilitation von Hüft-TEP Patienten verfolgt. Bitte kreuzen Sie auf der Skala von 1(höchste Priorität) bis 5(niedrigste Priorität) an.

	**1**	**2**	**3**	**4**	**5**
a. Verbesserung des Gleichgewichts	☐	☐	☐	☐	☐
b. Behandlung von muskulären Dysbalancen	☐	☐	☐	☐	☐
c. Verbesserung der Beweglichkeit	☐	☐	☐	☐	☐
d. Wiederherstellung des funktionalen Gangbildes	☐	☐	☐	☐	☐
e. Alltagsaktivität verbessern	☐	☐	☐	☐	☐
f. Rumpfstabilität	☐	☐	☐	☐	☐
g. Schmerzreduktion/ Schmerzfreiheit	☐	☐	☐	☐	☐
h. Kräftigung der hüftumgreifenden Muskulatur	☐	☐	☐	☐	☐

3.3. Wie sollte Ihrer Einschätzung nach **15 Tage postoperativ** das subjektive Belastungsempfinden in der medizinischen Trainingstherapie bei Krafttraining der unteren Extremitäten auf einer Skala zwischen 6 und 20 (Borg Skala) durchschnittlich sein?

	6	☐
sehr sehr leicht	7	☐
	8	☐
sehr leicht	9	☐
	10	☐
leicht	11	☐
	12	☐
mittel	13	☐
	14	☐
schwer	15	☐
	16	☐
sehr schwer	17	☐
	18	☐
sehr sehr schwer	19	☐
	20	☐

3.4. Wie sollte Ihrer Einschätzung nach **3 Monate postoperativ** das subjektive Belastungsempfinden in der medizinischen Trainingstherapie bei Krafttraining der unteren Extremitäten auf einer Skala zwischen 6 und 20 (Borg Skala) durchschnittlich sein?

	6	☐
sehr sehr leicht	7	☐
	8	☐
sehr leicht	9	☐
	10	☐
leicht	11	☐
	12	☐
mittel	13	☐
	14	☐
schwer	15	☐
	16	☐
sehr schwer	17	☐
	18	☐
sehr sehr schwer	19	☐
	20	☐

4. Fragen zur Einschätzung der Gelenkbelastung

4.1. Bitte schätzen Sie die Gelenkbelastung der Hüfte, von 1 (sehr geringe Belastung) bis 10 (maximale Belastung) bei folgenden Übungen/Bewegungen ein.

		1	2	3	4	5	6	7	8	9	10
a.	Ergometer- geringer Tretwiderstand,	☐	☐	☐	☐	☐	☐	☐	☐	☐	☐
b.	Ergometer- hoher Tretwiderstand	☐	☐	☐	☐	☐	☐	☐	☐	☐	☐
c.	Gehen 4km/h	☐	☐	☐	☐	☐	☐	☐	☐	☐	☐
d.	Einbeinstand	☐	☐	☐	☐	☐	☐	☐	☐	☐	☐
e.	Sitzen	☐	☐	☐	☐	☐	☐	☐	☐	☐	☐
f.	Aufstehen vom Stuhl	☐	☐	☐	☐	☐	☐	☐	☐	☐	☐
g.	Schulterbrücke / Bridging	☐	☐	☐	☐	☐	☐	☐	☐	☐	☐
h.	Abduktion in Seitlage	☐	☐	☐	☐	☐	☐	☐	☐	☐	☐

Vielen Dank für die Teilnahme an der Befragung.
