# Supplementary material for: Agreements and disagreements in exercise therapy prescriptions after hip replacement among rehabilitation professionals: a multicenter survey
Source: BMC Musculoskelet Disord. 2015 Aug 5;16:185. doi: 10.1186/s12891-015-0646-7 (PMC4526287; doi:10.1186/s12891-015-0646-7)
Supplement: Additional file 2: — Translated questionnaire (English). (PDF 157 kb) [file 12891_2015_646_MOESM2_ESM.pdf]

Questionnaire on exercise therapy prescriptions after hip replacement

Dear participant,
This questionnaire is designed to identify personal beliefs and institutional guidelines for exercise therapy treatment after hip replacement. We kindly ask you to answer the following questions. Your answers will help us to identify current standards of post-operative exercise therapy and the need of treatment guidelines. All information will be treated confidentially and anonymously. If you are uncertain regarding the most appropriate answer, please select the answer that you think is the closest to what you feel.
Thank you very much for supporting us.

1. Personal details (Please tick as appropriate or complete)

1.1. Which occupational group do you belong to?

a. Surgeon ☐
b. Orthopaedic physician ☐
c. Rehabilitation physician ☐
d. Exercise therapist ☐
e. Physiotherapist ☐
f. Other (please state) ………………………………………

1.2. How many years of professional experience do you have? ………………

1.3. In which of the following type of facilities do you work?

a. Outpatient rehabilitation clinics ☐
b. Acute inpatient hospital ☐
c. Inpatient rehabilitation clinics ☐
d. Physiotherapy practice ☐
e. Other (please state) ……………………………………..

1.4. How many patients with total hip replacement (THR) do you treat (roundabout, average) in a month?

…………..

2. <u>General questions about rehabilitation care and exercise therapy after total hip replacement</u> (Please tick as appropriate or complete)

2.1. What do you think influences the postoperative rehabilitation treatment? (Please rate according to the scale: 1 (unimportant), 5 (very important), multiple answers possible)

	1	2	3	4	5
a. Type of prosthesis (cemented/uncemented)	☐	☐	☐	☐	☐
b. Primary or secondary hip replacement	☐	☐	☐	☐	☐
c. Quality of surgery	☐	☐	☐	☐	☐
d. Course of surgery	☐	☐	☐	☐	☐
e. Constitution of the patient	☐	☐	☐	☐	☐
Further possible factors (please name):					
……………………………………….	☐	☐	☐	☐	☐
……………………………………….	☐	☐	☐	☐	☐
……………………………………….	☐	☐	☐	☐	☐
……………………………………….	☐	☐	☐	☐	☐

2.2. In your estimation, what is the optimal time to start full weightbearing after replacement surgery with a **cemented** prosthesis without any complications?

**0-5 days**	**6-10 days**	**11-20 days**	**21-30 days**	**5-6 weeks**	**7-8 weeks**	**more than 8 weeks**
☐	☐	☐	☐	☐	☐	☐

2.3. In your estimation, what is the optimal time to start full weightbearing after replacement surgery with an **uncemented** prosthesis without any complications?

**0-5 days**	**6-10 days**	**11-20 days**	**21-30 days**	**5-6 weeks**	**7-8 weeks**	**more than 8 weeks**
☐	☐	☐	☐	☐	☐	☐

2.4. In your estimation, what is the optimal time to start resistance training after replacement surgery with a **cemented** prosthesis without any complications?

**1-7 days post-operative**	**2-3 weeks post-operative**	**4-5 weeks post-operative**	**6-7 weeks post-operative**	**more than 8 weeks post-operative**
☐	☐	☐	☐	☐

2.5. In your estimation, what is the optimal time to start resistance training after replacement surgery with an **uncemented** prosthesis without any complications?

**1-7 days post-operative**	**2-3 weeks post-operative**	**4-5 weeks post-operative**	**6-7 weeks post-operative**	**more than 8 weeks post-operative**
☐	☐	☐	☐	☐

3. Questions on key exercise therapy components and rehabilitation objectives

3.1. What key exercise therapy components are used in your facility-specific standard rehabilitation protocol following hip replacement? (Please rate the activities according to the scale: 1 (highest priority), 5 (lowest priority))

		**1**	**2**	**3**	**4**	**5**
a.	Gym exercises	☐	☐	☐	☐	☐
b.	Continuous passive motion	☐	☐	☐	☐	☐
c.	Neuromuscular / sensorimotor training	☐	☐	☐	☐	☐
d.	Stretching	☐	☐	☐	☐	☐
e.	Water exercises	☐	☐	☐	☐	☐
f.	Gait training	☐	☐	☐	☐	☐
g.	Stair climbing	☐	☐	☐	☐	☐
h.	Ergometer cycling	☐	☐	☐	☐	☐
i.	Walking exercises	☐	☐	☐	☐	☐
j.	Manual therapy	☐	☐	☐	☐	☐
k.	Individual physiotherapy	☐	☐	☐	☐	☐
l.	Group exercises	☐	☐	☐	☐	☐

3.2. What are the overall objectives for the rehabilitation of hip replacement patients in your facility? (Please rate the activities according to the scale: 1 (highest priority), 5 (lowest priority))

		**1**	**2**	**3**	**4**	**5**
a.	Improving balance control	☐	☐	☐	☐	☐
b.	Reducing muscular imbalances	☐	☐	☐	☐	☐
c.	Improving mobility	☐	☐	☐	☐	☐
d.	Restoring functional gait patterns	☐	☐	☐	☐	☐
e.	Recovery of activities of daily living	☐	☐	☐	☐	☐
f.	Improving core stability	☐	☐	☐	☐	☐
g.	Pain reduction / freedom of pain	☐	☐	☐	☐	☐
h.	Strengthening of hip muscles	☐	☐	☐	☐	☐

3.3. In your estimation, what is the optimal strength training intensity (perceived exertion) at **15 days postoperatively** on a scale between 6-20 (Borg Scale)?

	6	☐
very, very light	7	☐
	8	☐
very light	9	☐
	10	☐
fairly light	11	☐
	12	☐
somewhat hard	13	☐
	14	☐
hard	15	☐
	16	☐
very hard	17	☐
	18	☐
very, very hard	19	☐
	20	☐

3.4. In your estimation, what is the optimal strength training intensity (perceived exertion) at **3 months postoperatively** on a scale between 6-20 (Borg Scale)?

	6	☐
very, very light	7	☐
	8	☐
very light	9	☐
	10	☐
fairly light	11	☐
	12	☐
somewhat hard	13	☐
	14	☐
hard	15	☐
	16	☐
very hard	17	☐
	18	☐
very, very hard	19	☐
	20	☐

4. Questions to assess the hip joint load during exercising

4.1. Please rate the joint load on the hip during the following exercises on a scale from 1 (very low load) to 10 (maximum load).

		1	2	3	4	5	6	7	8	9	10
a.	Ergometer cycling, low resistance	☐	☐	☐	☐	☐	☐	☐	☐	☐	☐
b.	Ergometer cycling, high resistance	☐	☐	☐	☐	☐	☐	☐	☐	☐	☐
c.	Walking 4km/h	☐	☐	☐	☐	☐	☐	☐	☐	☐	☐
d.	One-leg standing	☐	☐	☐	☐	☐	☐	☐	☐	☐	☐
e.	Sitting	☐	☐	☐	☐	☐	☐	☐	☐	☐	☐
f.	Chair rise	☐	☐	☐	☐	☐	☐	☐	☐	☐	☐
g.	Bridging	☐	☐	☐	☐	☐	☐	☐	☐	☐	☐
h.	Abduction in lateral position	☐	☐	☐	☐	☐	☐	☐	☐	☐	☐

Thank you for your participation.
